# Years of Life Lost (YLL) in Colombia 1998-2011: Overall and Avoidable Causes of Death Analysis

**DOI:** 10.1371/journal.pone.0125456

**Published:** 2015-05-05

**Authors:** Liliana Castillo-Rodríguez, Diana Díaz-Jiménez, Carlos Castañeda-Orjuela, Fernando De la Hoz-Restrepo

**Affiliations:** 1 Colombian National Health Observatory, Instituto Nacional de Salud, Bogotá, Colombia; 2 Instituto Nacional de Salud, Bogotá, Colombia; St Francis Hospital, UNITED STATES

## Abstract

**Objective:**

Estimate the Years of Life Lost (YLL) for overall and avoidable causes of death (CoD) in Colombia for the period 1998-2011.

**Methods:**

From the reported deaths to the Colombian mortality database during 1998-2011, we classified deaths from avoidable causes. With the reference life table of the Global Burden of Disease (GBD) 2010 study, we estimated the overall YLL and YLL due to avoidable causes. Calculations were performed with the difference between life expectancy and the age of death. Results are reported by group of cause of death, events, sex, year and department. Comparative analysis between number of deaths and YLL was carried out.

**Results:**

A total of 83,856,080 YLL were calculated in Colombia during period 1998-2011, 75.9% of them due to avoidable CoD. The year 2000 reported the highest number of missed YLL by both overall and avoidable CoD. The departments with the highest YLL rates were Caquetá, Guaviare, Arauca, Meta, and Risaralda. In men, intentional injuries and cardiovascular and circulatory diseases had the higher losses, while in women YLL were mainly due to cardiovascular and circulatory diseases.

**Conclusions:**

The public health priorities should focus on preventing the loss of YLL due to premature death and differentiated interventions by sex.

## Introduction

Decision-making in health is challenged to set, predict, and respond to the priorities of diseases prevention and control, ideally, based on summary measures of population health status [1, 2]. Deaths accountability is one of the simplest approaches to measure the health problems in a population. Mortality rates (MR) have been widely used to estimate the burden of disease and determine the relative importance of different causes of death [3–5].

MR play an important role in estimating the health status of populations and prioritizing the public health interventions, however, MR are generally not as sensitive to estimate the burden of premature mortality [6]. In fact, as most deaths occur in the elderly, MR are dominated by diseases of this population group [7]. The analysis of premature mortality involves the estimation of the average time that a person has left to live if death occurs prematurely (i.e. before her life expectancy). This estimation incorporate the age at which death occurs and not only the occurrence of the event [8].

The Years of Life Lost (YLL) is one way to measure the mortality impact, which gives higher weight to deaths at younger ages (premature mortality), and is useful in prioritizing public health interventions as elderly population deaths are less susceptible to decline. YLL, as summary measure of population health, are important to compare the relative importance of different diseases, to track differences in trends across countries and trends over time, and to provide a framework for evaluating the cost-effectiveness of new or better interventions [2]. The aim of this analysis was to estimate the YLL for both overall and avoidable Causes of Death (CoD) in Colombia during period 1998–2011.

## Methods

YLL for each death occurred in Colombia during period 1998–2011 were estimated. The National Bureau of Statistics (*Departamento Administrativo Nacional de Estadísticas—DANE*) is in charge to collect the certification of death register of all mortality case in the country. Death certificate is filled by health workers (a physician in most of cases) and includes variables about the individual identification and the basic CoD. *DANE* reviews the completeness and validity of the information and codifies the CoD as the International Classification Disease 10^th^ revision (CIE-10). *DANE* provided the Colombian mortality database for the present analysis. YLLs correspond to the number of years of life expectancy to the age of the death. The reference life expectancy for a single age was extracted from the life table of the Global Burden of Disease (GBD) 2010 Study from the University of Washington [9], and corresponds to a difference between that the expected age of death (*EAoD*) and the age of death (*AoD*).

A half cycle adjusting in the YLL estimation was performed to avoid the overestimation of the lost (i.e. a death occurred at the 11 months of any age). Deaths occurred after the first year of life included an adjusting factor (*k*) of 0.5 years; for population between two and 11 months, *k* was 0.42 years; and for children under two months *k* was zero, considering that the YLL loss was equivalent to life expectancy at birth. We did not use time discounts rate or adjustments for age weighting, according to the University of Washington recommendations [10].

$$YLL_{i}=EAoD_{i}-\left(AoD_{i}+k\right)$$ (1)

Where:

*YLL*: Years of Potential Life Lost of the individual _*i*_

*EAoD*: Expected Age of Death

*AoD*: Age of Death

*k*: half cycle adjustment factor according with the *AoD* (over one = 0.5; between two months and one year = 0.42, for less than two months = 0)

Avoidable deaths correspond to those deaths potentially preventable by both health system intervention and adjustment of policies pertaining to public health; the classification in Colombia was developed for the National Health Observatory and is published elsewhere [11]. To report the results the disease classification of the 2010 GBD study was implemented. There are 3 main groups: communicable, maternal, perinatal and nutritional diseases (group I); non-communicable diseases (group II); and injuries (group III); these groups are divided into 22 categories and 236 subcategories. Garbage code redistribution according with S1 Table was carried out.

We reported the estimated total annual YLL for both overall and avoidable CoD by sex and Colombian departments (States). In the analysis by department, deaths with no information for this variable were excluded. For overall CoD the analysis was carried out by residence’s department, except for dengue, malaria, and injuries, which were carried out by occurrence’s department. Data were managed and analyzed in MS Excel 2010 and Stata 12.

## Results

In Colombia during period 1998–2011 a total of 83,856,080 YLL for overall CoD were estimated, 76% of them due to avoidable CoD Table 1. The average annual loss was 5,989,720 YLL (rate 141.3 YLLs per 1000 habitants), and 4,548,802 YLL (rate 107.5 per 1000 habitants) for overall and avoidable CoD, respectively. Year 2000 had the higher YLL for both overall and avoidable CoD, with a rate of 163.5 and 130.8 per 1000 habitants, respectively; and the lowest was 2011 with rates of 116.3 and 81.2 per 1000, respectively Table 1.

**Table 1:** YLL estimates for overall and avoidable causes of death by year.

Year	YLL for overall CoD		YLL for avoidable CoD	
	Total	Rate per 1000 habitants	Total	Rate per 1000 habitants
1998	6,078,622	155.1	4,884,699	124.7
1999	6,364,830	160.2	5,108,467	128.6
2000	6,588,808	163.5	5,270,910	130.8
2001	6,632,278	162.5	5,269,116	129.1
2002	6,536,241	158.2	5,160,182	124.9
2003	6,197,433	148.1	4,781,512	114.3
2004	5,954,821	140.5	4,552,901	107.5
2005	5,763,788	134.4	4,318,683	100.7
2006	5,767,529	132.9	4,262,399	98.2
2007	5,710,459	130.0	4,183,692	95.2
2008	5,643,678	127.0	4,101,507	92.3
2009	5,692,451	126.6	4,106,973	91.3
2010	5,570,462	122.4	3,942,282	86.6
2011	5,354,680	116.3	3,739,908	81.2
**Total**	**83,856,080**		**63,683,232**	

Colombia, 1998–2011. CoD: Causes of Death

For overall and avoidable CoD, men were the most affected population group. The 64.7 and 68.4% of YLL for overall, and avoidable CoD occurred in men, respectively. For the total YLL in men, 80.1% were due to avoidable CoD, while in women it represented the 67.6% Table 2. For both sexes, the YLL trend is declining during the period analyzed Table 2.

**Table 2:** YLL for overall and avoidable causes of death by year and sex.

Year	Sex	YLL for overall CoD		YLL for avoidable CoD	
		Total	Rate per 1000 habitants	Total	Rate per 1000 habitants
1998	Male	3,971,514	205.4	3,339,020	172.7
	Female	2,103,931	106.0	1,542,891	77.7
1999	Male	4,166,215	212.5	3,492,522	178.1
	Female	2,197,015	109.2	1,614,674	80.2
2000	Male	4,350,832	218.8	3,649,401	183.5
	Female	2,236,647	109.6	1,620,336	79.4
2001	Male	4,392,380	218.1	3,663,452	181.9
	Female	2,238,898	108.3	1,604,774	77.6
2002	Male	4,353,839	213.5	3,631,758	178.1
	Female	2,180,713	104.2	1,527,432	73.0
2003	Male	4,022,838	194.8	3,278,214	158.7
	Female	2,172,345	102.5	1,501,551	70.8
2004	Male	3,842,964	183.8	3,103,761	148.4
	Female	2,111,319	98.4	1,448,810	67.5
2005	Male	3,662,776	173.0	2,899,384	137.0
	Female	2,100,625	96.7	1,418,972	65.3
2006	Male	3,663,247	171.0	2,867,463	133.8
	Female	2,103,230	95.7	1,394,000	63.4
2007	Male	3,630,365	167.0	2,826,738	130.4
	Female	2,078,241	93.4	1,355,441	60.9
2008	Male	3,572,671	162.8	2,761,855	125.8
	Female	2,070,219	92.0	1,339,094	59.5
2009	Male	3,643,593	164.1	2,808,014	126.5
	Female	2,048,858	90.0	1,298,958	57.0
2010	Male	3,547,755	157.9	2,693,827	119.9
	Female	2,022,347	87.8	1,248,198	54.2
2011	Male	3,391,902	149.2	2,542,355	111.8
	Female	1,962,253	84.2	1,197,140	51.4
Total 1998–2011 in Males		54,212,891	184.1	43,557,766	147.9
Total 1998–2011 in Females		29,626,641	98.0	20,112,272	66.5

Colombia, 1998–2011. CoD: Causes of Death

Five of the 33 departments (States) contributed with 50% of the total YLL for both overall and avoidable CoD: Antioquia, Bogotá DC (Colombia’s capital city), Valle del Cauca, Cundinamarca, and Atlántico Table 3. The departments with less YLL were Guainía, Vaupés, Archipiélago of San Andrés, Providencia and Santa Catalina, Amazonas, and Vichada. Analyzing the relative loss, the top five departments with higher YLL rates per 1000 due to overall CoD were: Caquetá, Risaralda, Caldas, Valle del Cauca, and Quindío; and for avoidable CoD, Caquetá, Guaviare, Arauca, Meta, and Risaralda Table 3.

**Table 3:** YLL for overall and avoidable causes of death by department.

Department	YLL for overall causes of death	YLL avoidable death	YLL for overall causes death rate per 1000 hab.	YLL avoidable death rate per 1000 hab.
Caquetá	1,108,750	929,920	188.6	158.2
Guaviare	225,576	194,753	169.2	146.1
Arauca	550,926	469,264	171.0	145.6
Meta	1,883,292	1,526,518	173.5	140.6
Risaralda	2,260,559	1,736,022	180.5	138.6
Valle del Cauca	10,200,694	7,984,099	175.9	137.7
Quindío	1,310,766	985,145	175.6	132.0
Antioquia	13,274,744	10,421,089	168.1	132.0
Norte de Santander	2,904,417	2,277,504	167.6	131.4
Caldas	2,383,760	1,772,258	175.9	130.8
Vichada	111,535	91,979	143.9	118.7
Putumayo	591,067	508,794	137.0	117.9
Casanare	575,660	472,299	140.9	115.6
Huila	2,088,410	1,585,811	148.6	112.9
Cesar	1,694,060	1,403,603	134.6	111.5
Tolima	2,865,995	2,120,573	150.5	111.3
Cauca	2,477,878	1,896,069	140.2	107.3
Vaupés	68,423	56,589	126.2	104.4
Guainía	62,487	50,612	128.6	104.1
Magdalena	1,998,742	1,605,757	124.5	100.0
Amazonas	112,466	90,649	120.3	97.0
Santander	3,617,450	2,595,071	132.4	95.0
Atlántico	3,647,665	2,785,226	121.1	92.5
Boyacá	2,319,379	1,608,529	132.4	91.9
Chocó	706,333	581,222	111.4	91.7
Cundinamarca	3,931,350	2,746,858	124.4	86.9
La Guajira	941,137	810,949	100.3	86.4
Córdoba	2,182,121	1,705,642	106.8	83.5
Nariño	2,383,435	1,787,434	111.1	83.3
Bolívar	2,734,946	2,125,443	104.2	81.0
Bogotá, D.C.	11,037,600	7,580,816	116.3	79.8
Sucre	1,067,332	813,786	99.2	75.6
Archipiélago de San Andrés Providencia y Santa Catalina	89,680	68,971	91.3	70.2

Colombia, 1998–2011. Results sort by YLL avoidable death rate per 1000 habitants. hab: habitants

**Fig 1:**
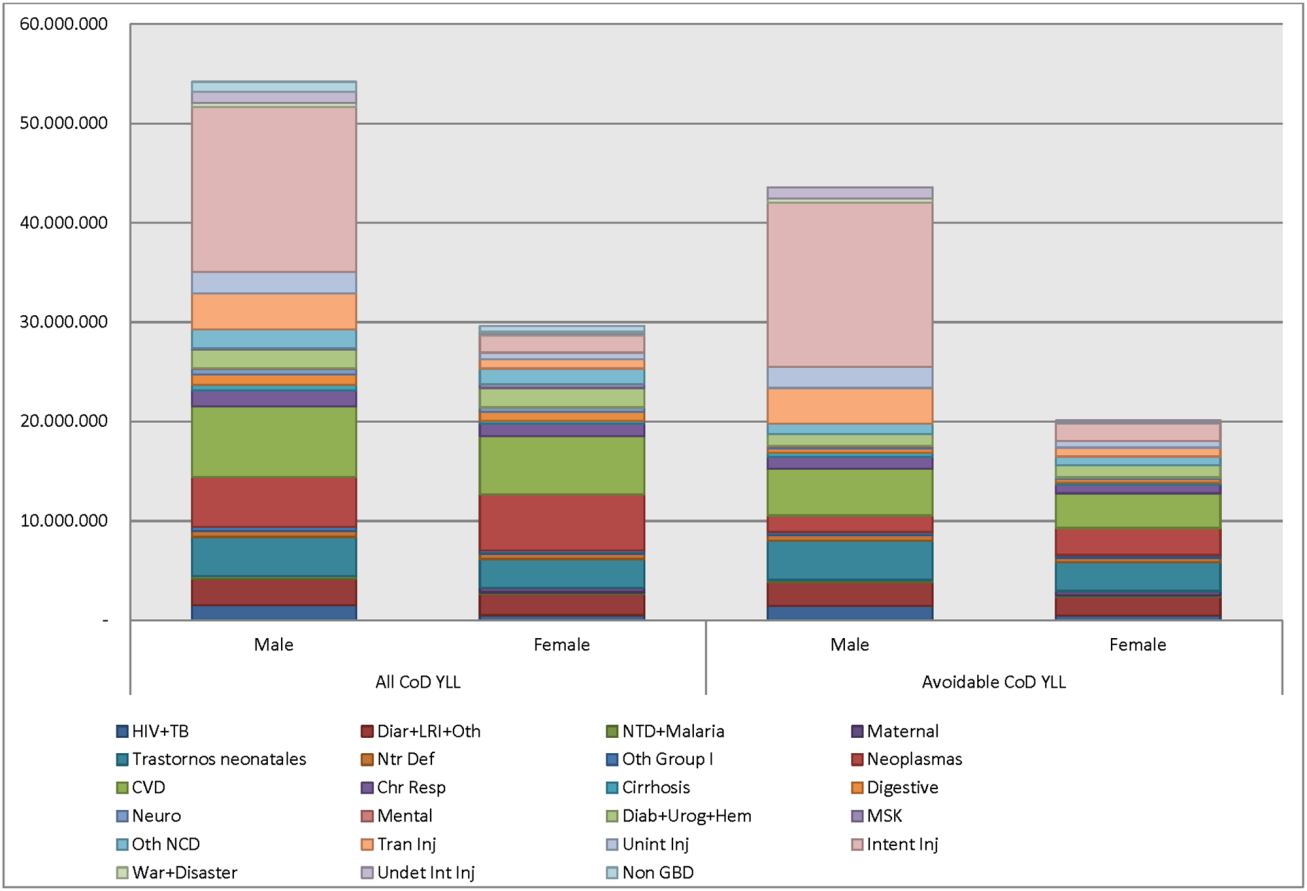
YLL for overall and avoidable causes of death by sex. Colombia, 1998–2011.

By main groups, for overall CoD during the period, 46% of YLL were due to non-communicable diseases, 33% to injuries, and 20% to communicable, maternal, neonatal and nutrition disorders; while for avoidable CoD 43% YLL were due to injury, 33% to non-communicable diseases, and 24% to communicable, maternal, neonatal and nutrition disorders. At the second aggregation level by sex, in males both overall and avoidable CoD YLL were mainly due to self-harm and interpersonal violence (intentional injuries) (31% and 38%, respectively), followed by cardiovascular and circulatory diseases (13% and 11%), and transit injuries (7% and 8%), while in women the higher YLL were due to cardiovascular and circulatory diseases (20% and 17%), and neoplasms (19% and 13%) (Fig 1). In both sexes, losses due to neonatal disorders also represented a significant YLL proportion of both overall and avoidable CoD (Fig 1).

**Fig 2:**
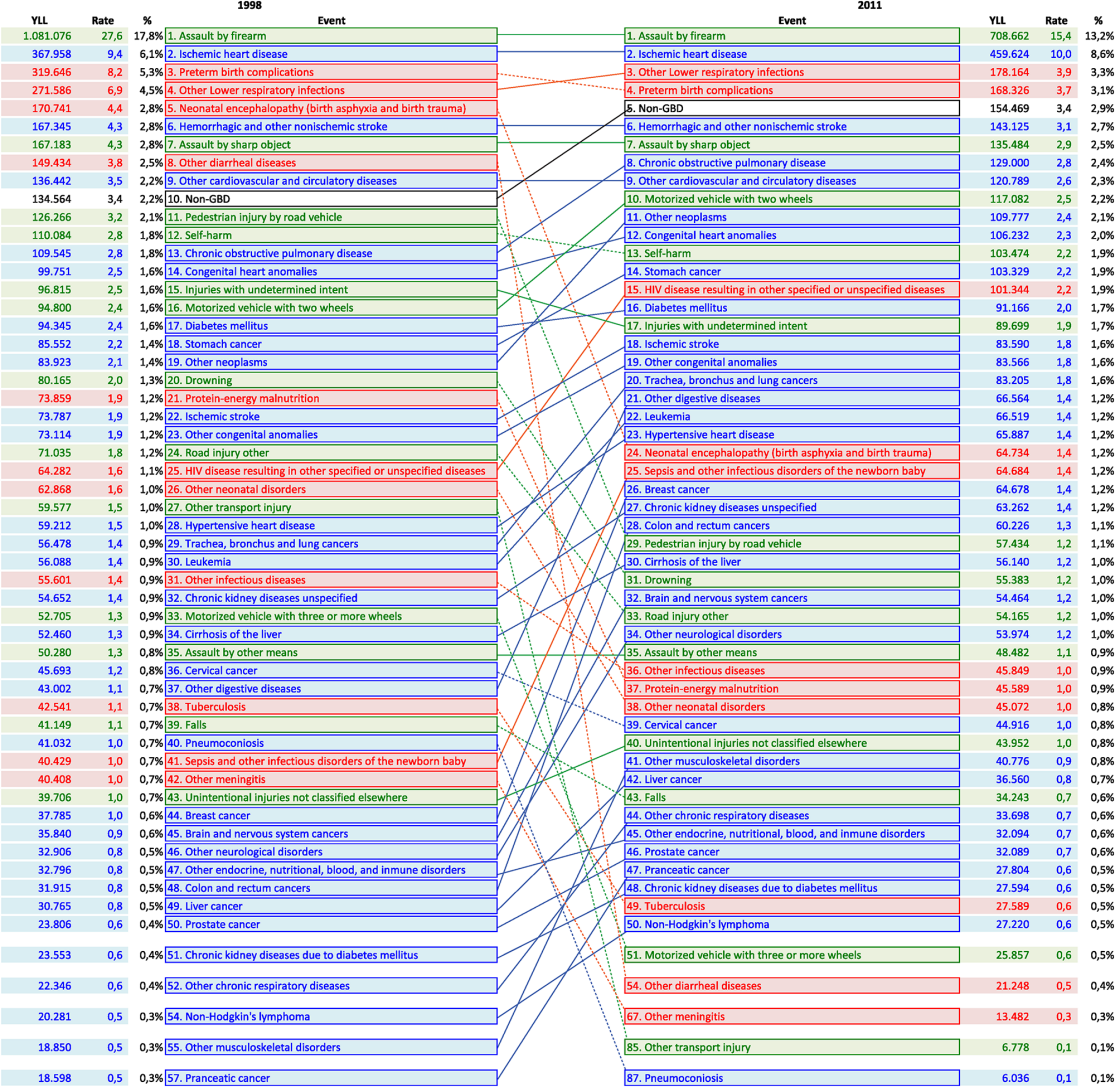
YLL for overall causes of death by event. Variation between 1998 and 2011, Colombia.

**Fig 3:**
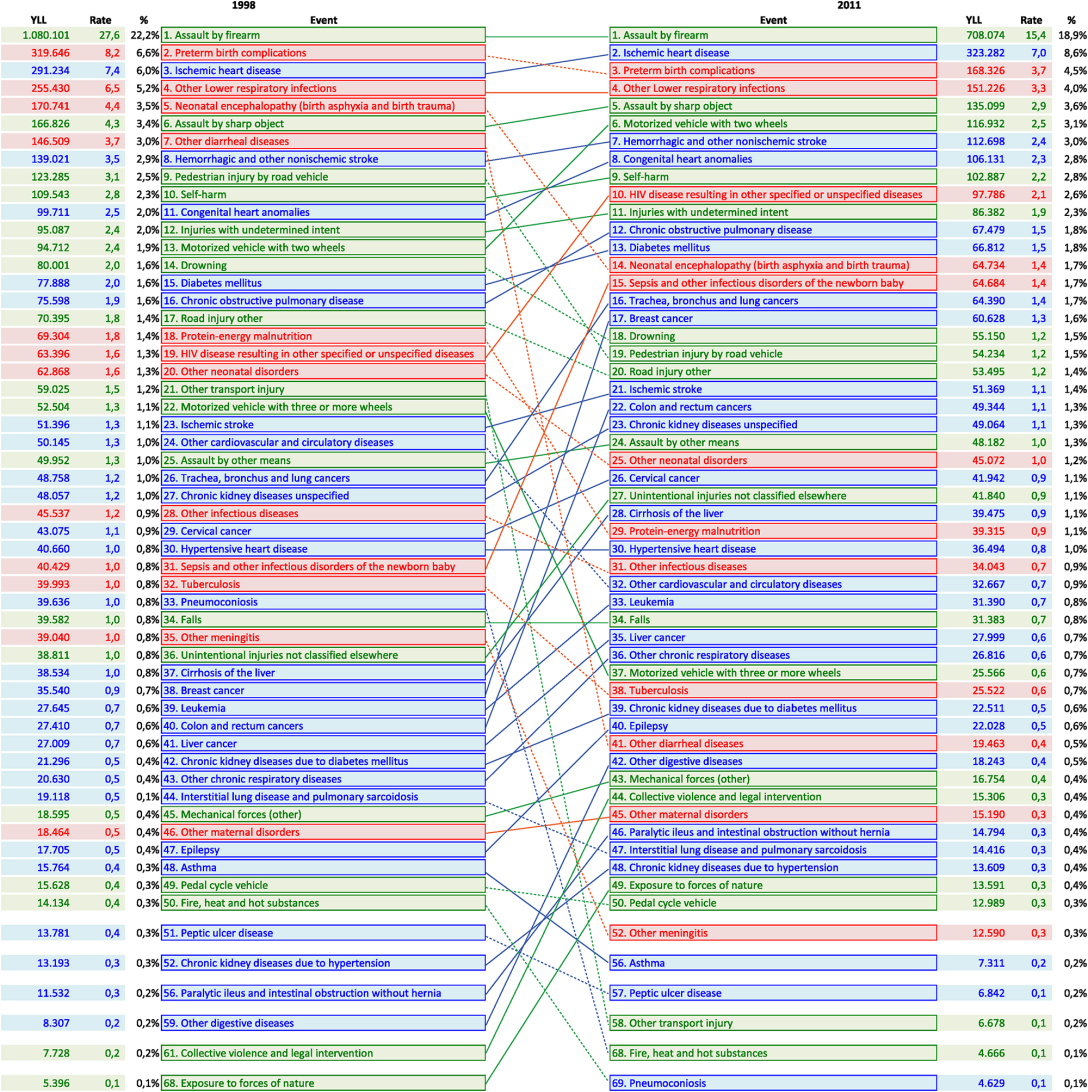
YLL by avoidable cause of death by event. Variation between 1998 and 2011, Colombia.

By events the analysis was performed comparing the first and later year of the period for both overall and avoidable CoD, Figs 2 and 3. Comparing overall CoD (Fig 2) it is noted that the assault by firearm was the leading cause of YLL in the two years, but the sharing in the total YLL was decreasing from 17.8% to 13.2%. Comparing the top 10 at the start and end of the period it is highlighted that Non-GBD Group raised from 10^th^ place in 1998 to 5^th^ place in 2011, and the absolute value increased from 134,564 YLL to 154,469 YLL; Chronic Obstructive Pulmonary Disease (COPD) changed from 13^th^ to 8^th^ place, varying from 109,545 to 129,000 YLL; and injuries in motorized vehicle with two wheels varied from 16^th^ to 10^th^ place rising from 94,800 to 117,082 YLL. Two events from Group I showed a significant reduction in terms of YLL: Neonatal encephalopathy, jumping from 5^th^ to 24^th^ place and other diarrheal diseases that moved from 8^th^ to 54^th^ place (Fig 2).

A YLL increase was observed for HIV disease resulting in other specified or unspecified disease varying from 64,282 in 1998 (25^th^ place) to 101,344 in 2011 (15^th^ place), other digestive diseases from 43,002 to 66,564 rising from 37^th^ to 21^th^ place, breast cancer from 37,785 to 64,678 changing from 44^th^ to 26^th^ place, and colon and rectum cancers from 31,915 to 60,226 moving from 48^th^ to 28^th^ place. In 2011 only two events from the group communicable, maternal, neonatal and nutritional disorders are among the YLL top 10, while 4 non-communicable diseases, and 3 injuries are in the top ten for overall CoD.

For the avoidable CoD the leading cause of YLL was also the assault by firearm for the start and end of the period, decreasing from 22.2% of total YLL to 19% in 2011, also with a decreasing in the absolute value of the YLL from 1,080,101 to 708,074 (Fig 3). It is highlighted the preterm birth complications of delivery that moved from the 2^nd^ place in 1998 to 3^rd^ in 2011 declining in 4.5% of its YLL. HIV disease resulting in other specified or unspecified disease was one of the top 10 events of avoidable mortality that report a high rose during the period going from 19^th^ to 10^th^ place (varying from 63,396 to 97,786 YLL). Other diarrheal diseases and neonatal encephalopathy decreased significantly, from 7^th^ and 5^th^ places to 41^th^ and 14^th^ places, respectively. Also decreased pedestrian injury by road vehicle (from 9^th^ to 19^th^ place). In 2011 the top 10 avoidable CoD, according with the YLL, were 4 injuries, 3 of the group I, and 3 non-communicable diseases.

## Discussion

This is the first Colombian national analysis performed in order to determine the impact of overall and avoidable mortality using YLL, the latter weighing the death for the time of occurrence [11]. In the literature, for Colombia only YLLs estimation to subnational scale or for particular events had been published [12–16]. During the period 1998–2011 in Colombia were estimated about 84 million YLL, 76% due to avoidable CoD, with trend to annual decrease in YLL rates for both overall and avoidable CoD.

We found absolute and relative YLL differences by department and sex. The largest losses are concentrated in larger departments, however in the analysis by YLL rates per 1000 population Caquetá, Risaralda and Caldas reported the highest avoidable YLL rates; suggesting YLL rates will be used whenever possible to evidence relatively important health problems in small population. By sex, YLL are higher in Colombia men, similar to the findings from other studies in Colombia [13–15] and in Italy [17, 18] and Spain [19]. Men is the most impacted population groups especially due to intentional injuries, while women is the cardiovascular and circulatory diseases.

The relative importance of non-communicable disease with 46% of YLL in overall CoD, is consistent with findings in other populations, although with a higher values close to 80% of total YLL [19, 20]. However, it is overcome by injuries when only YLL due to avoidable CoD were considered.

Very important differences between sexes are present, with significant implications for public health intervention with a differential approach. In men, for overall CoD, intentional injuries still in first place by YLL, but are cardiovascular and circulatory diseases, which have the highest number of deaths. The opposite occurs when analyzing the percentage proportion of avoidable CoD. In women, it is noteworthy that cardiovascular and circulatory diseases take first place in both the percentage ratio of YLL and deaths.

The events that occur early in life will bring more YLL and their intervention could have a higher avoiding in YLL in the Colombian population. Neonatal disorders and injuries of transport are relevant in both sexes, although the number of deaths is moderately they represent a significant loss of YLL and should be subject to preventive actions that reflect a significant impact on longevity.

These results could have an important impact on public health in Colombia, allowing to guide clear and specific interventions in preventive health care [16], and comparing YLL within the population over the years [21], referring to a normal life expectancy. The YLL is an important measure of population health status, and would be an approach to identify the interventions to implement with a positively affect the health of the population, even in avoidable CoD. The use of such indicators for prioritizing public health interventions requires simultaneous consideration of fairness and justice elements regarding population groups where YLL are not the best indicator of the health impact, but are relevant to advance in an agenda of better health reducing the burden of premature mortality. This study shows the utility of YLL as a measure of impact on life expectancy of the Colombian population; considering the assumption that to removing one of these specific causes that contributes significantly to the weight of the YLL, the population had not died from other causes [6].

This analysis has some limitations. First, there may be validity issues with the analysis depending of the correct identification of the basic CoD, in particular among the elderly people [22], however we use the clean and debugged official mortality database. Second, there are possibilities of classification errors by avoidable deaths, and the concept would need an in depth discussion, due to multiple available approaches. We focused in the avoidable deaths occurred before 75 years old, understand how the death that could not occur with an adequate health attention or a public policy implementation. Third, underreporting adjustment of mortality data was not performed and we only reported the analysis over the official deaths. The DANE coverage in Colombia is high, and the coverage had been identify in remote and small areas. However this analysis has the advantage that it is easy to do and only be requires death with the age of the events, with the population mortality rates or life tables [23].

## Conclusions

Cardiovascular and circulatory diseases and intentional injuries are the most relevant causes of YLL for both overall and avoidable death in Colombia. The YLL analysis is a useful tool for to define priorities and development public health policies that promote heath in universal and equitable way. Progress in the promotion and disease prevention can reduce the impact of events that could be avoidable and minimize treatment costs for the health system. Public health efforts and resources allocation in research should be more explicit in preventing premature death and to attain a substantial impact on life expectancy of the population. This type of analysis needs to be complemented with the assessment of the disabling effects of disease to estimate adjusted life years (DALYs), from the perspective of global burden of disease study, to promote informed health decisions.

## Supporting Information

S1 Table Recodification of garbage codes in the GBD 2010 groups. Redistribution of causes of death in ICD-10 garbage codes in the GBD study groups (Washington University), according with the WHO 2008-GBD groups.
(PDF)
