# Supplementary material for: Years of Life Lost (YLL) in Colombia 1998-2011: Overall and Avoidable Causes of Death Analysis
Source: PLoS One. 2015 May 5;10(5):e0125456. doi: 10.1371/journal.pone.0125456 (PMC4420276; doi:10.1371/journal.pone.0125456)
Supplement: S1 Table — Redistribution of causes of death in ICD-10 garbage codes in the GBD study groups (Washington University), according with the WHO 2008-GBD groups. (PDF) [file pone.0125456.s001.pdf]

Supporting Information

S1 Table. Recodification of garbage codes in the GBD 2010 groups.

Garbage codes	GBD codes	ICD-10 codes
**Septicaemia**	Other infectious diseases	A267, A327, A400, A401, A402, A403, A408, A409, A410, A411, A412, A413, A414, A415, A418, A419, A427, B377
	Other salmonella infections	A021
**Ill-defined codes from A00-B99**	Cirrhosis of the liver	B189
	Encephalitis	A86X
	Fungal skin diseases	B369
	Other diarrheal diseases	A049, A059, A079, A084
	Other infectious diseases	A209, A219, A229, A239, A244, A269, A279, A289, A319, A329, A429, A439, A449, A480, A490, A491, A492, A493, A498, A499, A669, A679, A699, A749, A819, A89X, A99X, B259, B279, B309, B340, B341, B342, B343, B344, B348, B349, B379, B382, B389, B399, B409, B419, B429, B439, B449, B459, B465, B479, B49X, B950, B951, B952, B953, B954, B955, B956, B957, B958, B960, B961, B962, B963, B964, B965, B966, B967, B968, B970, B971, B972, B973, B974, B975, B976, B977, B978
	Other meningitis	A879
	Other neglected tropical diseases	A689, A759, A779, A799, A94X
	Other sexually transmited diseases	A64X
	Viral skin diseases	B009
**Cardiogenic shock and other shock**	Other infectious diseases	A483
**B19, Unspecified viral hepatitis**	Other infectious diseases	B190, B199
**Pneumonitis**	Other chronic respiratory diseases	J677, J678, J679, J680
	Other infectious diseases	B250
	Pneumoconiosis	J690, J691, J698
**All unspecified**	Other infectious diseases	B589, B64X, B689, B820, B829, B89X

Garbage codes	GBD codes	ICD-10 codes
**parasitic diseases**	Other neglected tropical diseases	B719, B749, B789, B839
**Sequela of different diseases and injuries**	Gall bladder and bile duct disease	K870, K871
	Injuries with undetermined intent	Y872, Y899
	Ischemic stroke	I694, I698
	Other chronic respiratory diseases	J990, J991, J998
	Other digestive diseases	K238, K678, K910, K911, K912, K913, K914, K915, K918, K919, K938
	Other endocrine, nutritional, blood, and immune disorders	E68X, E890, E891, E892, E893, E894, E895, E896, E898, E899
	Other gynecological diseases	N950, N951, N952, N953, N958, N990, N991, N992, N993, N994, N995, N998, N999
	Other infectious diseases	B942, B948, B949, G09X
	Other musculoskeletal disorders	M032, M036, M074, M075, M076, M091, M092, M140, M141, M143, M144, M145, M146, M148, M360, M361, M362, M363, M364, M368, M490, M491, M492, M493, M494, M495, M498, M630, M631, M632, M633, M638, M738, M840, M841, M842, M901, M902, M903, M904, M905, M906, M907, M908, M960, M961, M962, M963, M964, M965, M966, M968
	Other neurological disorders	G130, G131, G132, G138, G530, G531, G532, G533, G550, G551, G552, G553, G558, G590, G598, G730, G731, G732, G733, G734, G735, G736, G737, G970, G971, G972, G978, G979, G990, G991, G992, G998
	Other nutritional deficiencies	E642, E643, E648, E649
	Other sense organ diseases	H590, H598
	Other skin and subcutaneous diseases	L998
	Other urinary diseases	N080, N081, N082, N084, N085, N088, N140, N141, N142, N143, N144, N160, N161, N162, N163, N164, N165, N168,

Garbage codes	GBD codes	ICD-10 codes
		N250, N251, N258, N259, N291, N298, N338, N350, N351, N370, N378
	Sense organ diseases	H950, H951, H958, H959
	Unintentional injuries other than transport injuries	Y859, Y86X
**B99, Other and unspecified bacterial and infectious diseases**	Other infectious diseases	B99X
**C14,1-.9, Other and ill- defined sites in the lip, oral cavity and pharynx**	Cancer of other part of pharynx and oropharinx	C142, C148
**C26,0-.9, Other and ill- defined sites in the lip, oral cavity and pharynx**	Other neoplasms	C260, C261, C268, C269
**C39,0-.9,Other and ill- defined sites in the lip, oral cavity and pharynx**	Other neoplasms	C390, C398, C399
**C55,0-.9, Uterus, part unspecified**	Uterine cáncer	C55X
**C57,9, Female genital organ unspecified**	Other neoplasms	C579
**C63,9, Male genital organ unspecified**	Other neoplasms	C639
**C68,9, Urinary organ unspecified**	Other neoplasms	C689
**C75,9, Endocrine gland unspecified**	Other neoplasms	C759
**C76,0-.9, Malignant neoplasm of other and ill- defined sites**	Other neoplasms	C760, C761, C762, C763, C764, C765, C767, C768
**C80,0-.9, Malignant neoplasm without specification of site**	Other neoplasms	C80X
**All unspecified site in situ carcinoma and unspecified site Neoplasm of**	Brain and nervous system cancers	D429, D432, D439
	Breast cancer	D059

Garbage codes	GBD codes	ICD-10 codes
**uncertain or unknown behavior**	Cervical cancer	D069
	Malignant melanoma of skin	D033, D039
	Non melanoma skin cancer	D043, D049
	Other neoplasms	D014, D019, D024, D073, D076, D091, D099, D379, D386, D399, D409, D419, D449
**Ill-defined codes from D10-D36,9**	Other neoplasms	D103, D105, D109, D126, D133, D144, D159, D179, D180, D181, D199, D229, D239, D269, D289, D299, D309, D316, D319, D329, D339, D359, D369
**DIC, cardiac arrest, acute respiratory failure and coma**	Other cardiovascular and circulatory diseases	I460, I461, I469
	Other endocrine, nutritional, blood, and immune disorders	D65X
	Other Lower respiratory infections	J960
**All encephalopathy and cerebral o edema**	Encephalitis	G050, G051, G052, G058
	Hemorrhagic and other nonischemic stroke	I64X
	Other neurological disorders	G92X, G930, G931, G932, G933, G934, G935, G936, G937, G938, G939, G948
	Other nutritional deficiencies	E512
**E85, Amyloidosis**	Other endocrine, nutritional, blood, and inmune disorders	E850, E851, E852, E853, E854, E858, E859
**All disorders of electrolyte & fluid balance**	Other endocrine, nutritional, blood, and inmune disorders	E86X, E870, E871, E872, E873, E874, E875, E876, E877, E878
**E889, Metabolic disorder unspecified**	Other endocrine, nutritional, blood, and inmune disorders	E889
**Ill-defined codes from F32-F99**	Other mental and behavioral disorders	F349, F39X, F459, F489, F519, F539, F609, F639, F649, F659, F669, F69X, F819, F849, F89X, F939, F949, F959, F989, F99X
**Ill-defined codes from G43-G58,9**	Other neurological disorders	G479, G509, G519, G529, G538, G542, G543, G544, G549, G569, G578, G589
**Different paralytic syndrome and palsy**	Other neurological disorders	G800, G801, G802, G803, G804, G808, G809, G810, G811, G819, G820, G821,

Garbage codes	GBD codes	ICD-10 codes
**syndrome**		G822, G823, G824, G825, G830, G831, G832, G833, G834, G838, G839
**All hydrocephalus**	Other neurological disorders	G910, G911, G912, G913, G918, G919, G940, G941, G942
**Ill-defined codes from H00-H99**	Cataracts	H281, H282, H288
	Other hearing loss	H919
	Other sense organ diseases	H019, H029, H030, H031, H038, H049, H059, H060, H061, H063, H103, H109, H119, H131, H132, H138, H159, H169, H179, H189, H190, H192, H193, H198, H209, H219, H279, H439, H449, H450, H451, H458, H470, H471, H477, H579, H580, H581, H588, H599, H609, H619, H699, H729, H739, H749, H750, H758, H809, H819, H82X, H839, H939
	Other vision loss	H309, H319, H320, H328, H349, H359, H539, H547
	Sense organ diseases	H220, H221, H228, H368, H480, H481, H488, H940, H948
**Hypertension**	Hypertensive heart disease	I10X, I150, I151, I152, I158, I159
**Pulmonary embolism**	Other cardiovascular and circulatory diseases	I260, I269
**I27, 1, Kyphoscoliotic heart disease**	Other cardiovascular and circulatory diseases	I271
**All ill-defined descriptions of heart disease**	Other cardiovascular and circulatory diseases	I279, I309, I319, I320, I321, I328, I349, I359, I369, I379, I38X, I390, I391, I392, I393, I394, I398, I510, I511, I512, I513, I514, I515, I516, I517, I518, I519
**I31, 2, Haemopericardium not classified elsewhere**	Other cardiovascular and circulatory diseases	I312
**I31, 3, Pericardial effusion (noninflammatory)**	Other cardiovascular and circulatory diseases	I313
**All cardiac conduction disorders**	Other cardiovascular and circulatory diseases	I440, I441, I442, I443, I444, I445, I446, I447, I450, I451, I452, I453, I454, I455, I456, I458, I459, I470, I471, I472, I479,

Garbage codes	GBD codes	ICD-10 codes
		I490, I491, I492, I493, I494, I495, I498, I499
**I50, Heart failure**	Other cardiovascular and circulatory diseases	I500, I501, I509
**Atherosclerosis**	Peripheral vascular disease	I700, I701, I709
**Embolism & thrombosis**	Other cardiovascular and circulatory diseases	I740, I741, I742, I743, I744, I745, I748, I749, I81X, I822, I823, I828, I829
**I99, Other and unspecified disorders of circulatory system**	Other cardiovascular and circulatory diseases	I99X
**Ill-defined codes from J30-J35,9**	Other chronic respiratory diseases	J304, J329, J339, J359
**J80, Adult respiratory distress syndrome**	Interstitial lung disease and pulmonary sarcoidosis	J80X
**J81, Pulmonary oedema**	Interstitial lung disease and pulmonary sarcoidosis	J81X
**Thorax and pleural problem**	Other chronic respiratory diseases	J90X, J91X, J920, J929, J930, J931, J938, J939, J940, J941, J942, J948, J949
	Other Lower respiratory infections	J860, J869
**J98, 9, Respiratory disorder unspecified**	Other Lower respiratory infections	J961, J969, J981, J982, J983, J989
**Ill-defined codes from K00-K14,9**	Other digestive diseases	K009, K039, K049, K069, K074, K079, K089, K099, K109, K119, K149
**K65, Peritonitis**	Other digestive diseases	K650, K658, K659
**Unspecified liver disease**	Cirrhosis of the liver	K769
**GI signs and syptoms**	Other digestive diseases	K920, K921
**K92,9, Disease of digestive system unspecified**	Other digestive diseases	K929
**Ill-defined codes from L01-L98, 9**	Other skin and subcutaneous diseases	L109, L119, L129, L139, L14X, L309, L439, L449, L45X, L519, L539, L540, L548, L559, L569, L589, L599, L609, L620, L628, L649, L659, L669, L679, L689, L719, L729, L739, L749, L759, L819, L859, L86X, L879, L909, L919, L929, L949, L959, L989

Garbage codes	GBD codes	ICD-10 codes
**Ill-defined codes from M09-M99**	Other musculoskeletal disorders	M098, M119, M130, M139, M206, M219, M229, M239, M249, M259, M319, M329, M339, M349, M359, M402, M405, M419, M429, M439, M464, M609, M619, M629, M659, M665, M679, M709, M719, M729, M759, M769, M779, M790, M792, M793, M799, M839, M849, M859, M879, M889, M899, M939, M949, M959, M969, M999
**Osteomyelitis**	Other musculoskeletal disorders	M462, M860, M861, M862, M863, M864, M865, M866, M868, M869
**N17-N19, Renal failure**	Chronic kidney diseases unspecified	N170, N171, N172, N178, N179, N180, N188, N189, N19X
**Ill-defined codes from N39,3-N97,8**	Other gynecological diseases	N609, N649, N759, N770, N771, N778, N829, N832, N839, N849, N859, N879, N889, N893, N899, N903, N909, N912, N915, N926, N939, N946, N949, N959
	Other urinary diseases	N399, N419, N429, N433, N489, N499, N510, N511, N512, N518
	Sexually transmited diseases excluding HIV	N709, N719, N732, N735, N739
**P96,9, Condition originating in the perinatal period unspecified**	Other neonatal disorders	P95X, P969
**Ill-defined codes from Q10-Q84,9**	Congenital heart anomalies	Q209, Q219, Q229, Q239, Q249, Q259, Q264, Q269, Q279, Q289
	Other congenital anomalies	Q129, Q139, Q149, Q159, Q169, Q179, Q189, Q309, Q319, Q339, Q349, Q399, Q403, Q409, Q419, Q429, Q438, Q439, Q459, Q519, Q529, Q539, Q549, Q559, Q563, Q564, Q602, Q605, Q613, Q619, Q639, Q649, Q652, Q655, Q659, Q669, Q699, Q709, Q719, Q729, Q730, Q738, Q749, Q759, Q769, Q779, Q789, Q799, Q809, Q819, Q829, Q839, Q849
**Q89,9, Congenital malformations unspecified**	Other congenital anomalies	Q899
**Q99,9, Chromosomal abnormality unspecified**	Other chromosomal abnormalities	Q999
**All unspecified transport and road**	Motorized vehicle with three or more wheels	V393, V399, V493, V499, V593, V598, V599, V693, V699, V793, V799

Garbage codes	GBD codes	ICD-10 codes
**injuries**	Motorized vehicle with two wheels	V293, V299
	Other transport injury	V819, V890, V891, V899, V99X
	Pedal cycle vehicle	V193, V199
	Pedestrian injury by road vehicle	V093, V099
	Road injury other	V809, V829, V892, V893
**X84, Intentional self-harm by unspecified means**	Self-harm and interpersonal violence	X840, X841, X842, X843, X844, X845, X846, X847, X848, X849
**Y09, Assault by unspec. means**	Assault by other means	Y090, Y091, Y092, Y093, Y094, Y095, Y096, Y097, Y098, Y099
**All undetermined intentional or unintentional**	Injuries with undetermined intent	Y100, Y101, Y102, Y103, Y104, Y105, Y106, Y107, Y108, Y109, Y110, Y111, Y112, Y113, Y114, Y115, Y116, Y117, Y118, Y119, Y120, Y121, Y122, Y123, Y124, Y125, Y126, Y127, Y128, Y129, Y130, Y131, Y132, Y133, Y134, Y135, Y136, Y137, Y138, Y139, Y140, Y141, Y142, Y143, Y144, Y145, Y146, Y147, Y148, Y149, Y150, Y151, Y152, Y153, Y154, Y155, Y156, Y157, Y158, Y160, Y161, Y162, Y163, Y164, Y165, Y166, Y167, Y168, Y169, Y170, Y171, Y172, Y173, Y174, Y175, Y176, Y177, Y178, Y179, Y180, Y181, Y182, Y183, Y184, Y185, Y186, Y187, Y188, Y189, Y190, Y191, Y192, Y193, Y194, Y195, Y196, Y197, Y198, Y199, Y200, Y201, Y202, Y203, Y204, Y205, Y206, Y207, Y208, Y209, Y210, Y211, Y212, Y213, Y214, Y215, Y216, Y217, Y218, Y219, Y220, Y221, Y222, Y223, Y224, Y225, Y226, Y227, Y228, Y229, Y230, Y231, Y232, Y233, Y234, Y235, Y236, Y237, Y238, Y239, Y240, Y241, Y242, Y243, Y244, Y245, Y246, Y247, Y248, Y249, Y250, Y251, Y252, Y253, Y254, Y255, Y256, Y257, Y258, Y259, Y260, Y261, Y262, Y263, Y264, Y265, Y266, Y267, Y268, Y269, Y270, Y271, Y272, Y273, Y274, Y275, Y276, Y277, Y278, Y279, Y280, Y281, Y282, Y283, Y284, Y285, Y286, Y287, Y288, Y289, Y290, Y291, Y292, Y293, Y294, Y295, Y296, Y297, Y298,

Garbage codes	GBD codes	ICD-10 codes
		Y299, Y300, Y301, Y302, Y303, Y304, Y305, Y306, Y307, Y308, Y309, Y310, Y311, Y312, Y313, Y314, Y315, Y316, Y317, Y318, Y319, Y320, Y321, Y322, Y323, Y324, Y325, Y326, Y327, Y328, Y329, Y330, Y331, Y332, Y333, Y334, Y335, Y336, Y337, Y337, Y338, Y339, Y340, Y341, Y342, Y343, Y344, Y345, Y346, Y347, Y348, Y349
